# Cold-Sprayed Ni and NdFeB-Al Powders Recovery and Reuse

**DOI:** 10.3390/ma18215000

**Published:** 2025-11-01

**Authors:** Jean-Michel Lamarre, Alexandre Nascimento, Cindy Charbonneau, Luc Pouliot, Fabrice Bernier

**Affiliations:** 1 National Research Council Canada, 75 Boul. de Mortagne, Boucherville, QC J4B 6Y4, Canada; cindy.charbonneau@cnrc-nrc.gc.ca (C.C.); fabrice.bernier@cnrc-nrc.gc.ca (F.B.); 2 Polycontrols Technologies Inc., 3650 Boul. Matte, Brossard, QC J4Y 2Z, Canada; alexandre.nascimento@polycontrols.com (A.N.); luc.pouliot@polycontrols.com (L.P.)

**Keywords:** cold spray, magnets, powder recovery, Nickel, NdFeB, sustainability

## Abstract

As cold spray additive manufacturing matures, significant efforts are being made to develop spray conditions for more challenging materials, thereby expanding the technology’s range of applications. One main challenge while using commercially available equipment is that, even under optimized conditions, deposition efficiency remains low for some materials. Powder particles that do not adhere are wasted, which can severely affect the process economics, especially in a mass production context and/or when expensive feedstocks are used. Powder recovery and reuse is a logical solution to mitigate this problem, yet few studies evaluate its feasibility and its impact on powder characteristics and ultimately coating performance. In this work, powder recovery was investigated for two cases: a Ni powder and a NdFeB-Al powder mix, used respectively for repair applications and for the fabrication of permanent magnets. A prototype recovery system was built, achieving a recovery efficiency of up to 75%. The powders were recovered after up to four spray runs, and their morphology and size distribution were characterized. The magnetic properties of both powders and coatings were evaluated using hysteresis measurements. Although the process affects the particle size distribution and their magnetic properties, powders remain suitable for re-deposition for both materials. In particular, it was shown that NdFeB-Al mix maintains 97% of its initial magnetic performance under industrial operating conditions.

## 1. Introduction

Increasing worldwide environmental targets are leading manufacturers to adopt more sustainable solutions and fabrication processes. In that context, electrification is rapidly expanding, which is reshaping material requirements, straining global supply chains, and more generally, creating challenges in terms of resource availability. These constraints have prompted several government bodies, such as those of Canada and the USA, to establish critical material lists and launch programs aimed at finding solutions to mitigate problems associated with the availability of these materials.

Additive manufacturing is a fabrication technique that can help address material availability issues by reducing material waste during production. Indeed, it has been demonstrated that additive manufacturing can produce near net shape parts, thus reducing the scrap and waste typically associated with subtractive manufacturing processes such as traditional machining [1]. Among the various additive manufacturing techniques available, cold spray deposition offers several distinct advantages, including the capacity to process materials in solid-state form (i.e., without melting and with minimal oxidation), remarkably high deposition rates that can reach several kilograms per hour, and the production of coatings with particularly good mechanical properties [2,3].

While cold spray additive manufacturing can process several kilograms of material per hour, it can also result in significant material waste, as the deposition efficiency (DE), defined as the ratio of powder adhering to the part to the total powder used, can be suboptimal for certain material systems [4]. DE is a complex function of process parameters, substrate nature and geometry, as well as the physical and chemical characteristics of the sprayed powder [5,6,7]. The deposition of composite materials containing a hard and/or brittle phase may lead to particularly low DE, which can seriously limit the industrial viability of the cold spray process from an economic standpoint when processing expensive powders. In the context of limited availability of critical materials, wasting a significant portion of the sprayed material is unacceptable from economic, environmental, and socio-ethical perspectives.

Since increasing cold spray DE is not always technically feasible, as it is highly dependent on the inherent properties of the sprayed material, powder recovery and reuse can emerge as a promising strategy to reduce material waste. The idea of achieving 100% powder usage was identified as a potential advantage of cold spray in the early stage of its development. Indeed, Segall et al. [8] suggested in 1998 that all particles rebounding during cold spray could eventually be reused. However, as the technology evolved, only limited efforts have been devoted to studying powder recovery and reuse, and the literature on the topic remains scarce. To date, powder reuse has been investigated only for MCrAlY [9], copper [10], and, more recently, for tin [11].

Early results indicate that powder recovery is feasible, that the recovered powder is only moderately affected by the spray process, and that it can improve the process economics. Nevertheless, both Perry et al. [10] and Zarazua-Villalobos et al. [11] reported significant decreases in the recuperated powder DE. For example, the DE of recuperated copper dropped to 46% compared to 68% for the as-received powder [10]. This decrease was attributed to changes in the particle size distribution as the particles with the most suitable characteristics are deposited during the first spray step as well as to particle work hardening. A similar effect was observed for tin, where deposition efficiencies for as-received and recuperated powders were 44% and 32%, respectively [11]. For both materials, the DE of a blend of recuperated and fresh powders was higher than that of a fully recuperated powder, potentially due to interactions between softer and harder particles and the resulting coating compaction effect. In the case of tin, a small reduction in mean particle size was also observed and attributed to particle erosion and fragmentation. Notably, the cumulative effect of multiple recovery cycles has not yet been investigated in the literature.

In this paper, two powders, NdFeB and Ni, containing elements listed as critical or near-critical by the US Department of Energy [12], were selected for investigation. The aim of the experiments was to study the effect of multiple spray-recovery cycles on the characteristics of these powders. Both powders are expensive (as of 2025) and are used in high value-added applications. The advantage of spraying NdFeB-Al composite powders for permanent magnets has been previously demonstrated, including for the fabrication of electric motor rotor prototypes using cold spray additive manufacturing [13,14]. Ni alloys, on the other hand, are widely used in cold spray aerospace repair applications [15]. Both powders present vastly different mechanical properties and are thus expected to respond differently to particle impact. Indeed, NdFeB powder is brittle, has limited deformability, and is consequently sprayed in combination with a softer metallic binder such as aluminum. In contrast, Ni is ductile and can be readily deposited using standard cold spray parameters [16,17]. Furthermore, NdFeB magnetic properties are sensitive to phase degradation and oxidation at high temperature, making them susceptible to modification during successive spray operations [18].

This paper presents results on the effects of recovery and reuse on the characteristics of both powders and the coatings produced from them. Specifically, the influence of up to four spray-recovery cycles was evaluated in terms of powder morphology and particle size distribution and compared to the original powders. In addition, coatings produced from these recuperated powders were characterized, and the results are discussed in relation to the intrinsic properties of each material. This paper evaluates the feasibility of recovering powders, with a focus on their magnetic properties, within the context of multiple recovery and reuse steps for sustainable industrial applications.

## 2. Methodology

### 2.1. Feedstock Preparation

Nickel (Amperit 176.068, Höganäs, Sweden), aluminum (H5, Valimet, Stockton, CA, USA), and NdFeB (MQFP-B, Neo-Magnequench, Toronto, ON, Canada) were used in this study. Table 1 summarizes the powder size distributions and fabrication methods for each material. Prior to spraying, the NdFeB powder was mechanically mixed with Al powder for 1 h in a 90 wt.%-10 wt.% ratio. Aluminum was added to promote bonding between magnetic particles during deposition [13].

### 2.2. Cold Spray Deposition

Cold spray deposition was carried out using a PCS-100 system (Plasma Giken Co., Saitama, Japan). The Ni powder and NdFeB-Al powder mix were sprayed using the parameters summarized in Table 2. The NdFeB-Al spray parameters were optimized in previous work [13], while Ni parameters were optimized to produce dense coatings at the same temperature. For both materials, a target feed rate of approximately 20 g/min was used. The substrate for Ni deposition was a 76 mm × 76 mm × 3 mm grit-blasted mild steel plate (ASTM A36). For NdFeB-Al deposition, a 76 mm × 76 mm × 6 mm as-received cold-rolled Al6061 plate was used.

### 2.3. Powder Recovery

**Figure 1:**
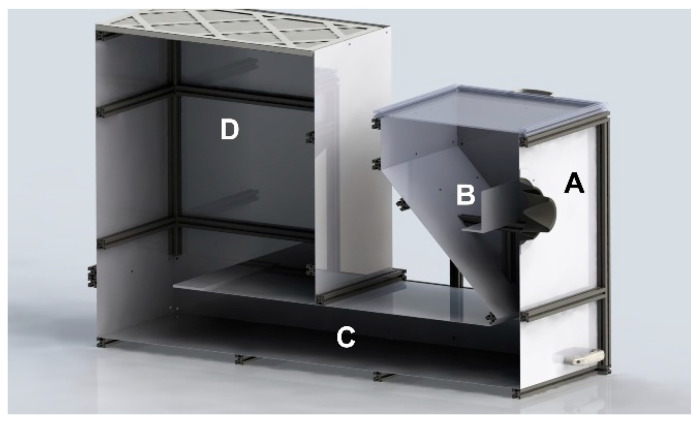
Schematic view of the recovery system consisting of four zones: (A) the gun entrance, (B) the substrate holder, (C) the gas deceleration area, and (D) the exit chimney and filter.

Powder recovery during cold spray is uncommon in a R&D environment due to several challenges: (1) direct recovery from the dust collector is neither simple nor efficient; (2) contamination from other powders and materials may occur; and (3) handling fine metallic particles poses explosion hazards. Previous studies have recovered powder by lining the cold spray chamber with aluminum foil [11] or using a custom powder reclamation chamber [9]. An approach similar to the latter was adopted in this work: a custom recovery chamber was designed and assembled, as illustrated in Figure 1, using extruded aluminum profiles and galvanized mild steel plates. The chamber consists of four zones: A—cold spray gun entrance, B—substrate holder area, C—gas deceleration area, and D—the exit chimney and filter. Deposition occurs in zone B, where the substrate is positioned perpendicular to the gun’s main spray axis. The back wall is placed more than 10 cm behind the substrate and angled to minimize unwanted deposition.

Multiple system iterations were developed and tested to achieve >50% recovery efficiency, considered a benchmark for industrial feasibility. This also ensures that the recovered powder is representative of the non-adhered fraction and provides sufficient material for re-spray experiments. The recovery chamber efficiency was improved using several means: (1) A high temperature rubber gasket sealing the gun nozzle to the chamber entrance was installed. The gasket is flexible enough to allow to lateral gun movement for deposition. (2) High-temperature silicon and gaskets were used to minimize gas leaks at the steel panels and aluminum profiles interfaces. (3) Internal panels were installed to lengthen the gas path and increase the travel time in zone C. (4) An exit chimney-like feature was installed in zone D. (5) An industrial-grade filter was installed at the chamber outlet to capture particles.

The recovery process involved spraying the powders using the parameters in Table 2 inside the recovery chamber and collecting the substrate plates and recovered powders at regular intervals for characterization. Recovered powders were collected from zones C and D of the recovery chamber using a fine brush and a dust tray. An industrial solution for collection of the powders would still need to be developed. Powders were labeled by generation number (e.g., Generation 2 = sprayed and recovered twice; AR-0 = as-received powder was labeled as 0 since it has not been sprayed nor recovered). Coatings were labeled based on the powder generation that was used in their fabrication. As an example, a coating labeled generation 3 was sprayed with a powder of generation 3.

For the NdFeB-Al mix, due to differences in material properties and deposition behavior, Al powder was added after each recovery cycle, prior to the next spray step, to maintain the original mass fractions of each component. The quantity of Al added was determined from powder density measurements, carried out using a gas pycnometer (AccuPyc 1330, Micromeritics, Norcross, GA, USA). Powder was added to maintain a constant mix density consistent with the initial composition of 90 wt.% NdFeB and 10 wt.% Al.

### 2.4. Characterization

Several tests were performed to characterize the powders, including density, particle size, morphology, hardness, and magnetic performance. These tests were carried out on both the as-received powder and after each recovery cycle to evaluate the effect of successive spray runs on powder characteristics.

Particle size distributions were measured by laser diffraction (LS I3 320, Beckman Coulter, Indianapolis, IN, USA). Particle Vickers microhardness was obtained from polished resin-mounted samples using a 10-gf load (MMT-X7B, Matsuzawa Co., Tokyo, Japan). Powder magnetic properties, namely, remanence (ability to retain magnetization) and coercivity (resistance to demagnetization under a magnetic field), were measured with a Permagraph L (Magnet Physics, Cologne, Germany). For these measurements, a known quantity of powder was mixed into liquid epoxy, which was subsequently cured before testing.

Particle morphology was examined by scanning electron microscope (Apreo 2 15 kV, Thermofisher, Waltham, MA, USA). Magnification was selected to meet the ISO 9276-6 requirements for the minimum number of pixels required for accurate analysis of the smallest particles [19]. Image analysis followed ISO 13322-1 [20] using a dedicated software (Clemex Vision PE version 8.0.197, Clemex Technologies Inc., Longueuil, QC, Canada). Image quality was enhanced by applying a pruning function to remove single-pixel wide peaks and valleys, followed by segmentation and automatic bridge removal to separate touching particles [21]. Among the measured morphological parameters (area, convexity, aspect ratio, compactness, roundness, roughness, extent), the aspect ratio was selected as the most representative for assessing the impact of recovery on morphological features.

Coatings produced at each iteration were characterized in terms of DE (i.e., the ratio of mass retained relative to total sprayed mass over a substrate), porosity, and magnetic properties (remanence and coercivity). The NdFeB volume fraction was determined by SEM image analysis and pycnometry. The same equipment and measurements methodologies were used for both powders and coatings.

## 3. Results

The recovery system described in Section 2.3 was first used to spray the as-received Ni powder and NdFeB-Al powder mix, producing recovered powders of Generation 1. Each subsequent spray cycle used the recovered powders from the previous generation, up to production of powder of Generation 4.

To evaluate the system’s performance, the masses of sprayed, deposited, and recovered powders were measured. The sprayed powder mass was obtained by weighing the powder in the hopper before and after spray. The deposited mass was determined from the substrate weight change. The impinging mass was calculated from the effective feed rate and the robot’s movement parameters. From these values, the system/powder deposition efficiency (DE), recovery efficiency (RE), and total efficiency (TE) were calculated using the following equations:

(1) $$DE\left(\%\right)=\frac{deposited\;mass}{impinging\;mass}$$

(2) $$RE\left(\%\right)=\frac{recuperated\;mass}{sprayed\;mass-deposited\;mass}$$

(3) $$TE\left(\%\right)=\frac{recuperated\;mass+deposited\;mass}{sprayed\;mass}$$

In this study, the traditional definition of the DE was adopted, i.e., DE represents the mass ratio of deposited particles to that of impinging particles. RE measures the mass ratio of the non-adhered powder that is recovered. TE quantifies the fraction of the sprayed powder that is either deposited or recovered. These three ratios are linked together by Equation (4).

(4) $$TE\left(\%\right)=DE\left(\%\right)+RE\left(\%\right)\;(1-DE\left(\%\right))$$

**Figure 2:**
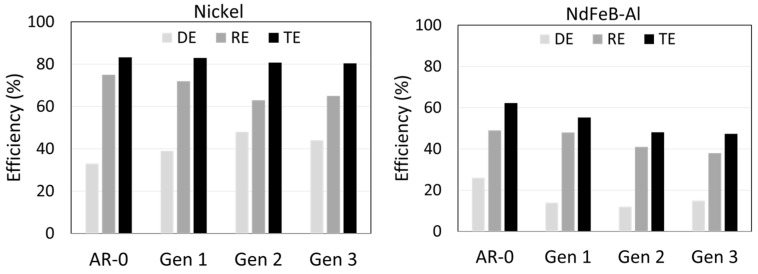
Deposition efficiency (DE), recovery efficiency (RE) and total efficiency (TE) for Ni (**left**) and NdFeB-Al (**right**) powders as a function of powder generation. Data are shown in the above graph for the sprays of the as-received powder (AR-0), as well as for Generations 1 to 3. Each spray produces the next generation of recovered powders as follows: (i) spray AR-0 → recovery of Generation 1, (ii) spray of Generation 1 → recovery of Generation 2, (iii) spray of Generation 2 → recovery of Generation 3, (iv) spray of Generation 3 → recovery of Generation 4.

The results for Ni and NdFeB-Al recovered powders are presented in Figure 2. For Ni, the as-received powder (AR-0) had a DE of 33%, which increased to 48% for Generation 2 before slightly decreasing to 44% for Generation 3. The RE for Ni was initially close to 75%, decreasing to ≈ 65% for Generations 2 and 3. The TE remained relatively stable, from 83% (AR-0) to 80% (Generation 3).

On the other hand, for NdFeB-Al, the AR-0 DE was 26%, dropping sharply to 14%, 12%, and 15% for Generations 1, 2, and 3, respectively. The RE was below 50% for AR-0 and declined to 38% by Generation 3. Consequently, the TE decreased from 62% (AR-0) to 47% (Generation 3). It is worth noting that producing enough Generation 4 powder for testing requires spraying a significant amount of AR-0 powder due to the system’s limited RE and the fraction of powder that is successfully deposited. This is particularly true for NdFeB-Al, where the RE is below 50%. Using the data in Figure 2 and Equations (1)–(4), it was calculated that 12 kg of NdFeB-Al and 8 kg of Ni are needed to obtain 200 g of Generation 4 powder, enough for characterization. This limitation strongly restricts the number of generations that can be studied in a laboratory setting. However, spraying up to Generation 4 is sufficient to establish clear trends.

### 3.1. Nickel

**Figure 3:**
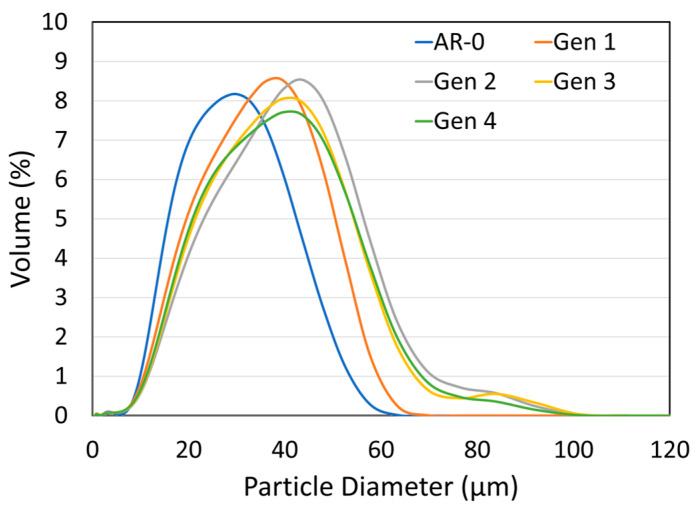
Effect of the spray-recovery cycles on the particle size distribution for the as-received Ni powder (AR-0) and recovered powders (Generations 1 to 4).

The particle size distribution of the as-received Ni powder and the recovered powders from the subsequent generations are shown and compared in Figure 3. The corresponding *d*_10_, *d*_50_, and *d*_90_ values are summarized in Table 3. The as-received Ni powder has a distribution mostly comprising particles between 14 µm (*d*_10_) and 43 µm (*d*_90_). After each spray-recovery cycle, the particle size increases as can be seen, for example, by comparing the size distribution of Generations 1 and 2. This is particularly evident in the *d*_90_, which rises from 43 µm for the as-received powder (AR-0) to 49 µm and 57 µm for Generations 1 and 2, respectively. For Generations 2, 3, and 4, the *d*_90_ stabilizes between 55 and 57 µm.

**Figure 4:**
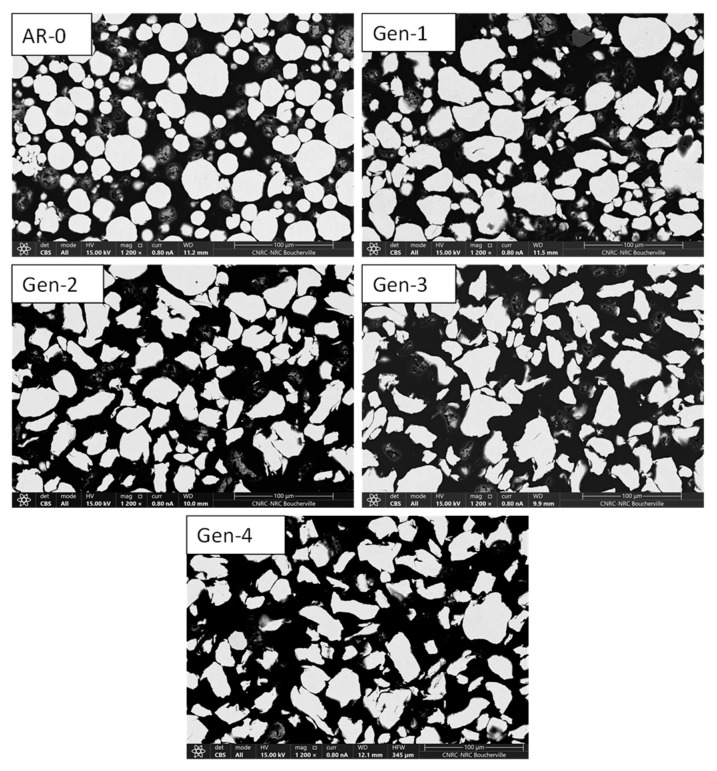
SEM cross-sections images showing the evolution of Ni powder morphology for as-received (AR-0) and recovered powders (Generations 1 to 4). The powder becomes more angular with each spray-recovery cycle. Powder of Generation 4 is mostly angular and comprises some internal porosities.

SEM micrographs comparing the morphologies of the as-received and recovered powders highlight that the AR-0 particles are generally more spherical and of uniform composition (Figure 4). As the number of cycles increases, three notable observations can be made: (1) particles become significantly more angular which is visible by comparing powder AR-0, Generation 1, and Generation 4; (2) internal porosity becomes slightly more prevalent in recovered powders; and (3) there is no evidence of particle agglomeration or fusion after spraying.

**Figure 5:**
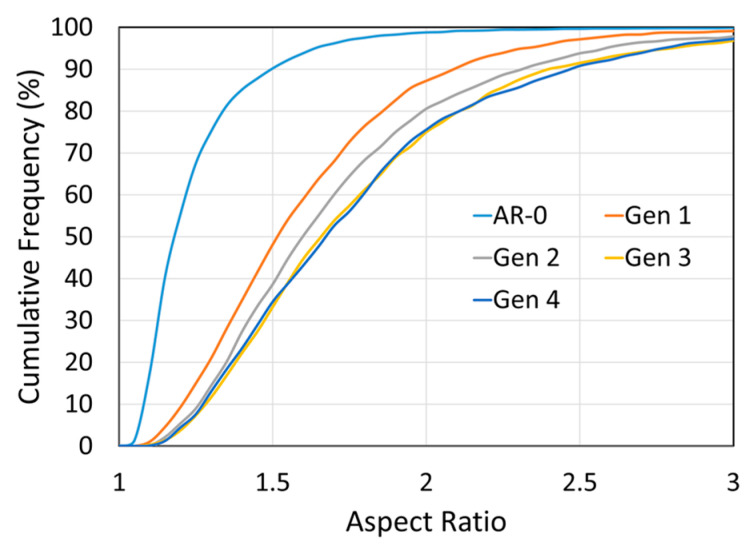
Effect of spray recovery cycles on Ni particle aspect ratio for as-received powder (AR-0) and recovered powders (Generations 1 to 4). The aspect ratio increases after the first cycle and stabilizes from Generation 3 onward.

Quantitative image analysis of 20 SEM images per generation was performed to characterize the evolution of the particle morphologies (see Section 2.4). The particle aspect ratio is defined as the ratio of the particle’s long axis to its short axis. For the as-received Ni powder, the light blue curve in Figure 5 shows that most particles are relatively equiaxed, with 70% having an aspect ratio below 1.3 and 90% below 1.5. A marked change occurs after the first spray-recovery cycle, with nearly 50% of particles exhibiting aspect ratios above 1.5, highlighting a clear morphological shift. As the generation number increases, the aspect ratio continues to rise slightly but stabilizes from Generations 3 to 4.

**Figure 6:**
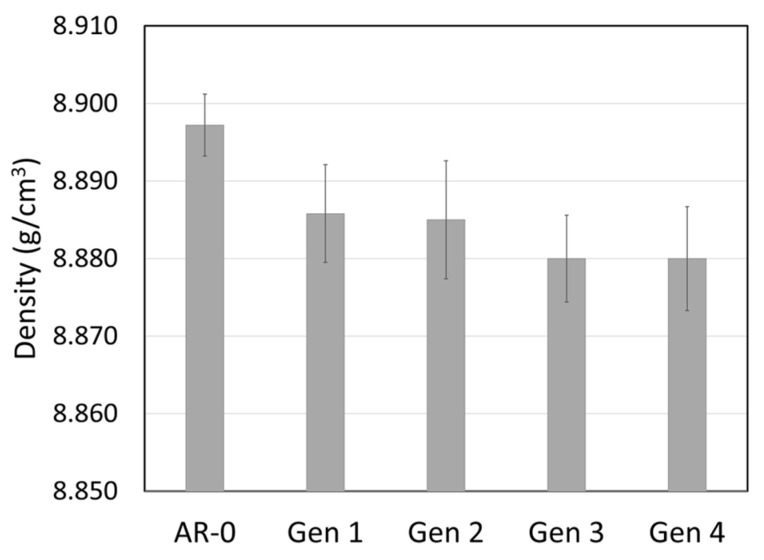
Effect of spray-recovery cycles on Ni powder density for the as-received powder (AR-0) and recovered powders (Generations 1 to 4).

**Figure 7:**
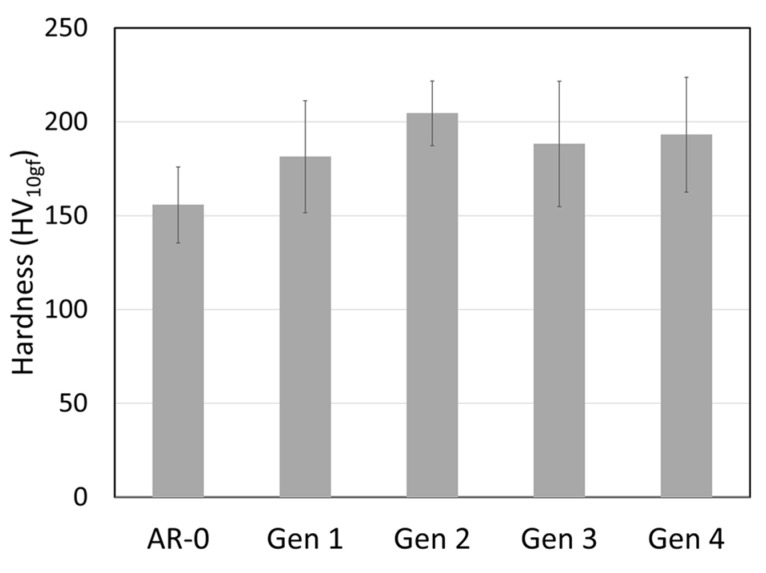
Effect of spray-recovery cycles on Ni powder hardness for the as-received powder (AR-0) and recovered powders (Generations 1 to 4).

Recovered Ni powders were also characterized for density and hardness (Figure 6 and Figure 7). Powder density decreases slightly with the number of cycles, while the hardness increases significantly from just above 150 HV for AR-0 to nearly 200 HV for Generations 2 to 4.

**Figure 8:**
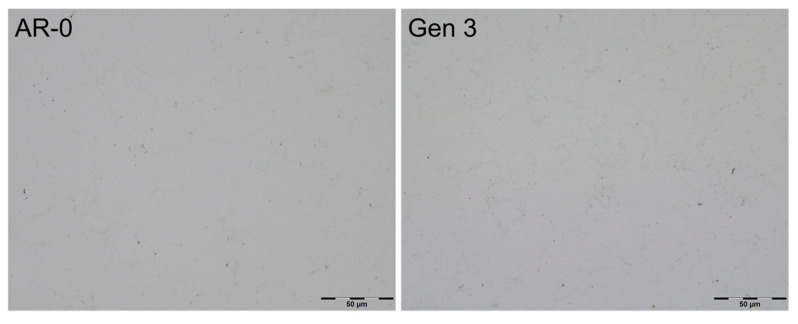
Metallography of Ni coatings produced from as-received powder (AR-0) and recovered powder (Generation 3). Generations 1 and 2 coatings were exhibiting very similar microstructures.

Coatings produced from all generations reached thicknesses of up to 13 mm without delamination. All coatings were dense, with no visual differences in color or surface roughness between generations. Metallography observations (Figure 8) show that coatings from AR-0 and Generation 3 powders are nearly identical, with minimal porosity and no significant defects. Coatings from Generations 1 and 2 displayed similar microstructures.

**Figure 9:**
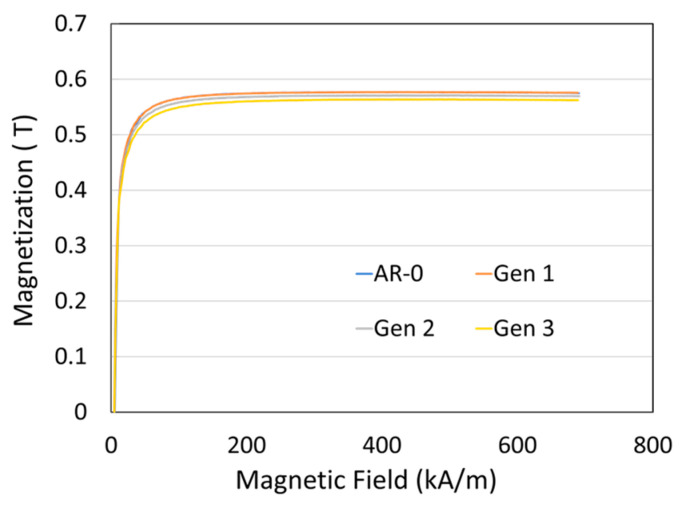
Effect of spray-recovery cycle on the magnetization curves of the Ni coatings produced from as-received powder (AR-0) and recovered powders (Generations 1 to 3).

The magnetic properties of the coatings are shown in Figure 9. All powder generations exhibited induction saturation values close to 0.57 T, with near-zero remanent magnetization once the magnetic field is removed.

### 3.2. NdFeB-Al

**Figure 10:**
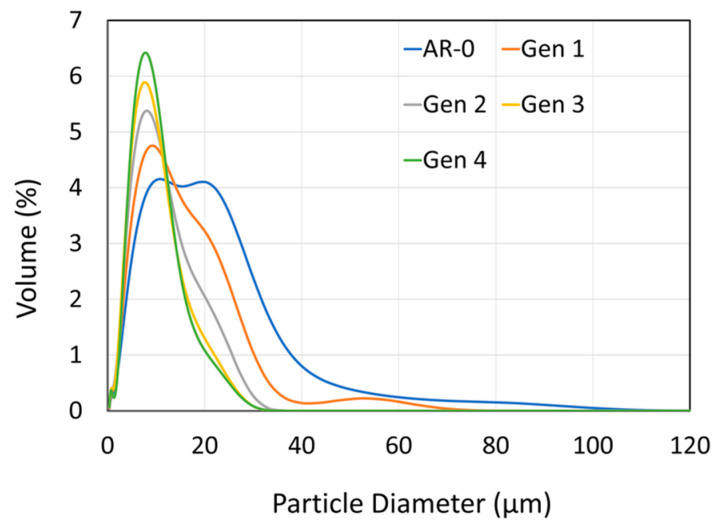
Effect of spray-recovery cycles on NdFeB-Al composite particle size distribution for the as-mixed powder (AR-0) and recovered powders (Generations 1 to 4).

The NdFeB-Al powder mix was sprayed and recovered using the same procedure as for the Ni powder. The particle size distributions for the as-mixed powder (AR-0) and recovered powders (Generations 1 to 4) are shown in Figure 10 and summarized in Table 4. The initial powder mix exhibits a double Gaussian distribution, corresponding to the coarser NdFeB particles and the finer Al particles.

A significant change in particle size distribution occurs with increasing spray-recovery cycles: the fraction of large particles (>20 microns) decreases steadily, while the proportion of fine particles increases. This trend is reflected in the *d*_90_, which decreases from 28.8 µm down to 14.5 µm between AR-0 and Generation 4. The *d*_50_ follows a similar trend, from 11.2 µm to 7.3 µm, while the *d*_10_ remains nearly constant.

**Figure 11:**
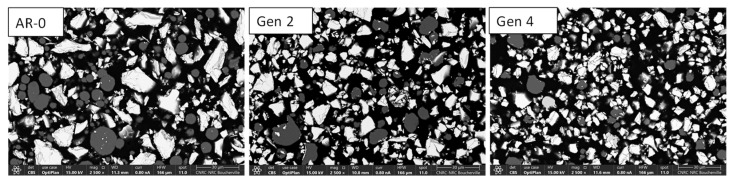
SEM cross-sections images of NdFeB-Al powder for as-mixed powder (AR-0) and recovered powders (Generations 2 and 4).

SEM cross-section images (Figure 11) show that AR-0 consists of irregularly shaped NdFeB particles (light grey, rough edges) mixed with smaller, more spherical Al particles (darker grey). Both materials are dense and uniform in composition. One can readily observe a decrease both in aluminum and NdFeB particle size when comparing the micrographs of the Generation 2 powder with that of the AR-0 powder. By Generation 4, NdFeB particles appear less angular and more spherical compared to the AR-0 powder.

**Figure 12:**
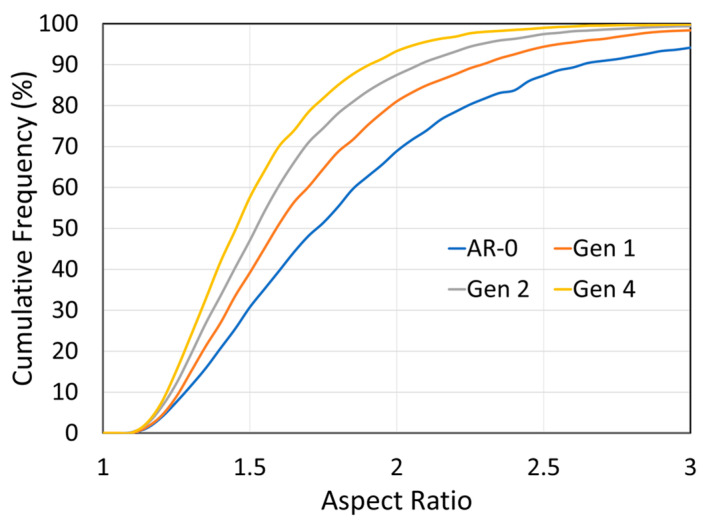
Effect of spray-recovery cycles on NdFeB-Al particles’ aspect ratio for as-mixed powder (AR-0) and recovered powders (Generations 1,2 and 4). The particles’ aspect ratio is increasing with each spray-recovery cycle.

Image analysis results for the aspect ratio are shown in Figure 12. In contrast to the Ni powder, the aspect ratio decreases with increasing spray-recovery cycles, which is consistent with the SEM observation that particles become more spherical. This trend shows no sign of saturation up to Generation 4, suggesting it would likely continue beyond.

**Figure 13:**
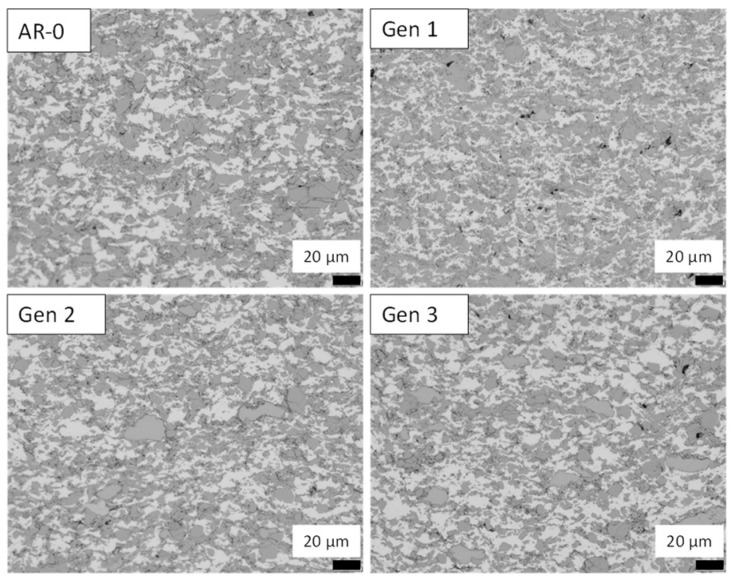
Optical micrographs of NdFeB-Al coatings produced from as-mixed powder (AR-0) and recovered powders (Generations 1 to 3).

Coating cross-sections for AR-0 and Generation 1 to 3 are presented in Figure 13. As in the powder cross-sections, the NdFeB particles appear in dark grey, while the Al phase appears lighter. For all generations, the coatings exhibit good intersplat cohesion and low porosity, although the latter is slightly higher for coatings produced from re-sprayed powders compared with the as-mixed material. For example, a slightly higher void content can be observed when comparing AR-0 and Generation 1 micrographs. In both cases, the porosity remains below 1%.

**Figure 14:**
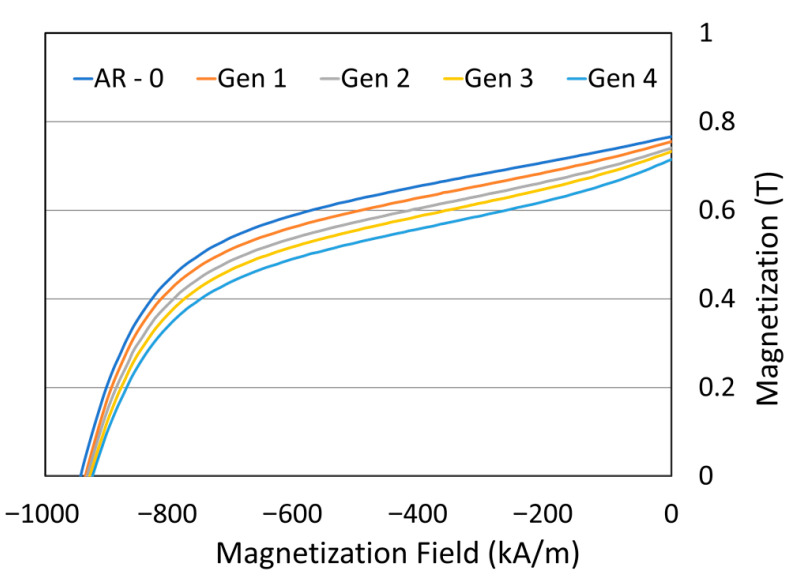
Effect spray-recovery cycles on demagnetization curves for NdFeB-Al powders (top) for as-mixed powder (AR-0) and recovered powders (Generations 1 to 4).

Magnetic properties of the powders are shown in Figure 14. The second quadrant of the demagnetization curve is presented, where the magnetic induction at zero field corresponds to the remanence, and the *x*-axis intercept corresponds to the coercivity. Both remanence and coercivity show a slight decline with increasing generations, with the remanence decrease being more pronounced. Table 5 summarizes the magnetic properties as well as densities of the magnets produced with powders of different generations. Remanence decreases progressively with generation number.

## 4. Discussion

### 4.1. Recovery Efficiency

As calculated from Equation (2), the recovery efficiency (RE) of the system for the Ni powder (≈75%) was higher than that for the NdFeB-Al powder mixture (≈50%). This observation can be attributed mainly to differences in particle size and density. As shown in Figure 3 and Figure 10 and Table 3 and Table 4, the as-received Ni powder has a larger *d*_50_ of 26 µm, compared with 11 µm for the mix. Moreover, Ni powder has a higher density (8.9 g/cm^3^, Figure 6) than NdFeB (≈7.5 g/cm^3^) and significantly higher than Al (2.7 g/cm^3^). Both larger particle size and higher density increase particle settling velocity according to Stokes’ law, meaning heavier and larger particles are more likely to be collected. The filtering and deceleration mechanisms in zones C and D of the chamber (Figure 1) are likely more effective at capturing such particles. Improving RE for finer powders would require a more effective deceleration zone but would likely remain challenging. For both materials, RE decreased with increasing spray-recovery cycles, most likely due to changes in powder characteristics over successive impacts.

### 4.2. Recuperated Powder Characteristics

Figure 4 and Figure 11 show that particle morphology for both powders was significantly altered by the spray process. For Ni, the mean particle size increased after recovery (Figure 3). This can partly explain the system’s greater efficiency in recovering larger particles, leading to a relative reduction in the proportion of particles < 20 µm. Large Ni particles (>40–50 µm) may also have lower DE due to a higher critical velocity, causing them to rebound and be recovered [9,11,22]. However, this explanation is not fully consistent with the observed increase in DE over generations (Figure 2). Very large particles (>60 µm) appear from Generations 2 onward (Figure 3), possibly due to particle agglomeration (though this not evident in SEM images, Figure 4) or particle deformation (more likely). Elongated particles broaden the size distribution and may appear larger (or smaller) when measured by laser diffraction, depending on orientation.

Morphologically, Ni particles become progressively more angular with each spray-recovery cycle (Figure 4 and Figure 5), as reflected by the increase in particle aspect ratio. Particle deformation and flattening was also observed for soft Cu powder [10] and harder, but still deformable, MCrAlY powder [9]. The as-received powder is relatively spherical due to the gas-atomization process used in its production. Being metallic and ductile, Ni particles can undergo significant deformation upon impact, making them susceptible to shape changes. By Generation 4, a particle has experienced four impacts and is therefore likely to be heavily deformed in multiple directions. This deformation trend appears to stabilize after several cycles, as indicated by both SEM observations and aspect ratio measurements. The increased hardness of the latter-generation Ni powders may also contribute to this stabilization.

The hardness of the Ni powder increases by ≈30% with spray and recovery (Figure 7), likely due to strain hardening during impact with the substrate. This value is higher than the 10% increase reported for MCrAlY by Guo et al. [9]. The slight density decrease (≈0.2%, Figure 6) may be linked to the internal porosity observed in Generation 4 particles (Figure 4), but this reduction is likely too small to significantly affect spray behavior or the properties of the coatings produced from the recovered powders.

In contrast to the Ni results, the particle size of recovered NdFeB-Al decreases with each spray-recovery cycle (Figure 10). This might seem counterintuitive in light of the RE discussion in Section 4.1, but two factors can explain this trend. First, the fine, low-density Al particles are more strongly influenced by the bow shock effect [23] near the substrate and may therefore be overrepresented in the recovered powder. Second, NdFeB particles are brittle and can fracture upon impact, particularly when colliding with previously deposited NdFeB particles within the coating. This fragmentation produces smaller particles, explaining both the absence of particles larger than 30 µm after Generation 2 and the microstructural observations in Figure 11. Fracture also affects morphology. The as-received NdFeB particles are angular due to the crush-ribbon fabrication process. Being brittle, NdFeB particles undergo little to no plastic deformation upon impact and instead tend to fracture. Consequently, this produces smaller fragments which, statistically, are more spherical since impacts and fractures occur in random directions.

### 4.3. Coating Characteristics and Deposition Efficiency

Given the higher hardness of recovered Ni powders, one expects a more challenging spray process and reduced DE. However, micrographs (Figure 8) show that dense coatings were obtained for both as-received and Generation 3 powders. There is no visible microstructural evidence that the higher hardness of powder of Generations 2 and above led to degradation of the deposited coating properties and microstructure due to reduced deformation. Furthermore, coating density and quality remained constant despite significant change in powder characteristics.

Interestingly, DE increased for recovered Ni powders compared to the as-received powder, despite higher hardness. This is contrary to what was observed in previous powder recovery studies on copper and tin [10,11], where higher hardness was generally associated with reduced deposition efficiency. This atypical behavior for Ni may be related to the reduced fraction of fine particles, which are more susceptible to the bow shock, although this hypothesis was not experimentally confirmed. It is important to note that deposition efficiency depends on multiple factors beyond hardness, including particle size and shape, and experimental conditions such as gas temperature and pressure, as well as substrate material. Differences in these parameters between the present study and prior work on copper and tin may explain the observed deviation from trends reported in the literature.

The magnetic properties of Ni coatings (Generations 0–3) showed no significant variation, with a magnetic saturation of approximately 0.57 T close to that of pure wrought Ni (0.61 T) and consistent with dense, oxidation-free coatings. This somewhat lower value is reasonable considering the natural defect density of cold-sprayed materials. These microstructural and magnetic results indicate that Ni powders recovered multiple times can still produce coatings with adequate properties, although the increased hardness may influence mechanical performance and adhesion. The system’s total efficiency (TE) remained above 80% for all Ni generations, despite a relatively low DE and a recovery system that was not fully optimized for industrial operation.

Both as-received and recovered NdFeB-Al powders produced dense coatings, although porosity was slightly higher for the recovered powders (Figure 13), consistent with the density reductions observed for Generations 2 and 3 (Table 5). This reduction in density corresponds to a small decrease in the calculated volume fraction of NdFeB. DE dropped substantially after recovery for the NdFeB-Al mix (14% vs. 26%) compared to the as-received powder (Figure 2). Two possible explanations for this decrease are as follows: (1) the significant changes in particle size distribution and morphology and (2) altered mechanical behavior of the Al powder where, as observed in the case of Ni, deformation may affect deposition performance. Due to the small size and high ductility of Al particles, hardness could not be measured precisely. TE for NdFeB-Al mix decreased from 62% (AR-0) to 47% (Generation 3), which is relatively low from an industrial feasibility standpoint.

From a magnetic standpoint, coatings exhibited remanence values ranging from 0.39 to 0.48 T, with the lowest value obtained for the coating produced from Generation 3 powder. The reduction in magnetic properties can be attributed to two factors: (1) decreased remanence of the NdFeB powder feedstock (approximately 6% drop by generation 4) and (2) reduced coating density. This degradation in magnetic performance would likely worsen with additional spray-recovery cycles.

In a more realistic industrial production setting, the feedstock would consist of a mixture of fresh and recycled powders. Table 6 models a scenario in which fresh as-received powder is added after each spray run to compensate for powder losses which are including both deposited powder and powder not recovered by the collection system (this fraction corresponds to 1-TE). In this model, the DE is assumed to be low at 15%, similar to values observed for Generations 2–4, and the RE is set at 80%, a realistic target for a production-ready system. Under these conditions, 68% of the powder would be recycled and 32% would be fresh material added at each spray run.

**Figure 15:**
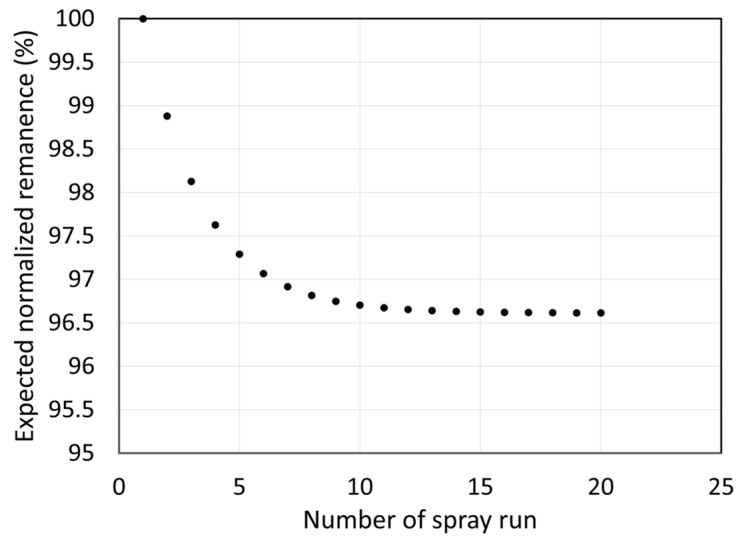
Expected normalized remanence of the powder used for a large number of sprays in a production scenario. The powder used from each spray run is obtained from a mix of recovered powder from the previous run and the addition of as-received powder.

Using these parameters and the measured remanence values for each generation, the “steady-state” production powder approximated by spray run 5 in Table 6 and composed of a mixture of powders from all previous generations would have a remanence equal to 97.4% of that of the as-received powder. Figure 15 shows the calculated evolution of remanence for powders including generations beyond 4, extrapolated from Table 6 data. A remanence decrease of 1.65% per generation was assumed, based on results for Generations 1–4. Calculations for a recycled powder containing material from theoretical Generations 1–20 indicate that the “steady-state” composition (32% fresh, 68% recycled from Generations 1–20) would retain 96.6% of the original powder’s remanence. This is a significant result: even with low DE and relatively fast powder degradation (1.65% per generation), a mixed feedstock containing a high volume fraction of recycled powder could still produce coatings with good magnetic properties. Given that NdFeB powders are particularly prone to thermal degradation, the same approach could yield even better results for other materials.

### 4.4. Next Steps Towards Industrialization

While the magnetic performance results are promising, several enhancements and additional measurements are required before the system can be considered for industrial application. The main areas for improvement include (1) increasing the system’s recovery efficiency (RE) and automating powder collection, (2) evaluating and quantifying potential contamination sources, (3) assessing the mechanical properties of the deposited materials, and (4) testing system performance with other materials.

(1) Recovery efficiency and powder collection—As discussed in Section 4.1, the RE for Ni powder is high; however, the value obtained for the NdFeB–Al composite is relatively low. Several strategies may be investigated, with the most promising being optimization of the deceleration area (Section C, Figure 1) through an increased number of internal panels or improved geometry. In parallel, the development of an automated powder collection system will be essential.
(2) Contamination assessment—Although the current recovery system has been tested with a single material, future studies should examine cross-contamination risks associated with multi-material use, as well as external contamination factors such as sealing gasket integrity.
(3) Mechanical property evaluation—Key cold spray properties, including adhesion and cohesion, were not measured in this study due to the limited powder quantities produced in later generations. These properties are likely to be influenced by the observed changes in particle morphology and hardness. For example, the distinctive DE effect noted for Ni could potentially be clarified through such measurements. Likewise, a detailed examination of NdFeB fragment structures may provide further insight into the observed decrease in magnetic properties and the underlying mechanisms.
(4) Testing with additional materials—Given the strong results for Ni and NdFeB–Al, extending the study to other materials would be valuable, particularly for further validating the conclusions drawn here.

## 5. Conclusions

The recovery and reuse of two high-value materials, Ni and NdFeB, was investigated for the cold spray process. The study showed that powder particles undergo significant changes in both shape and size distribution during successive spray and recovery cycles. The nature of these changes depends on their physical characteristics: soft metallic Ni powder became increasingly deformed and elongated after multiple spray operations, while harder, ceramic-like NdFeB particles tended to become more spherical. Ni powder exhibited a notable increase in hardness, which could potentially hinder the spray process; however, the microstructure of the deposited material remained of high quality even after four recovery cycles. Ni magnetic properties remained excellent throughout all spray runs at 93% of pure Ni saturation. The magnetic properties of the NdFeB powder were examined in detail, showing that a production feedstock composed of 32% fresh powder and 68% recuperated powder could retain 96.6% of the performance of fresh powder. The results indicate that powder recovery and reuse in cold spray is feasible and can produce coatings with properties that are acceptable for a wide range of applications.

## Figures and Tables

**Table 1:** Powder feedstock production methods and particle size distributions.

Powder	Production	*d*_10_	*d*_50_	*d*_90_
			(µm)	
Nickel, Amperit 176.068	Gas atomized	13	26	39
Al, H5	Gas atomized	4	9	15
NdFeB, MQFP-B	Crushed ribbon	4	16	26

**Table 2:** Cold spray deposition parameters for both materials.

Spray Parameters	Nickel	NdFeB-Al
Pressure (MPa)	4.9	4.9
Temperature (°C)	600	600
Process Gas	Nitrogen	Nitrogen
Feed Rate (g/min)	40 ± 4	24 ± 3
Raster Velocity (mm/s)	100	100
Raster Pitch (mm)	1	1
Standoff Distance (mm)	40	80
Substrate material	Steel	Al 6061

**Table 3:** Ni particle size distribution (*d*_10_, *d*_50_, and *d*_90_) for as-received powder (AR-0) and recovered powders (Generations 1 to 4).

Powder Generation	PSD by Volume (µm)		
	*d*_10_	*d*_50_	*d*_90_
AR-0	14	26	43
Gen 1	15	31	49
Gen 2	17	36	57
Gen 3	16	34	55
Gen 4	16	33	56

**Table 4:** NdFeB-Al composite particle size distribution for as-mixed powder (AR-0) and recovered powders (Generations 1 to 4).

Powder Generation	PSD by Volume (µm)		
	*d*_10_	*d*_50_	*d*_90_
AR-0	3	11.2	28.8
Gen 1	3	9.0	22.5
Gen 2	2.6	7.5	17.5
Gen 3	2.6	7.1	15.0
Gen 4	3.1	7.3	14.5

**Table 5:** Magnetic properties and density of NdFeB-Al magnets produced with recovered powders.

Processed Powder Generation	Density (g/cm^3^)	NdFeB Calculated Volume Fraction (%)	Magnet Remanence (T)
Gen 1	5.39	56	0.48
Gen 2	5.39	56	0.44
Gen 3	5.27	54	0.44
Gen 4	5.01	48	0.39

**Table 6:** Powder composition per spray run with added AR-0 powder accounting for deposited powder (DE 15%) and recovery efficiency (RE 80%). A total of 68% of the initial powder is recovered after each run. Mixed powder expected remanence is calculated from individual generation values (indicated in the title row).

Spray Run	AR 0 (0.765 T)	Gen 1 (0.755 T)	Gen 2 (0.740 T)	Gen 3 (0.730 T)	Gen 4 (0.715 T)	Remanence (T)
1	100	0	0	0	0	0.765
2	32	68	0	0	0	0.758
3	32	22	46	0	0	0.751
4	32	22	15	31	0	0.748
5	32	22	15	10	21	0.745

## Data Availability

The original contributions presented in this study are included in the article. Further inquiries can be directed to the corresponding author.
